# Protein Engineering and Drug Discovery: Importance, Methodologies, Challenges, and Prospects

**DOI:** 10.3390/biom15111628

**Published:** 2025-11-20

**Authors:** Ahmed Mohammed, Nasir A. Ibrahim, Nosiba S. Basher

**Affiliations:** 1 Department of Biotechnology, College of Life Sciences and Technology, Omdurman Islamic University, Omdurman 382, Sudan; 2 Biology Department, College of Science, Imam Mohammad Ibn Saud Islamic University (IMSIU), Riyadh 11623, Saudi Arabia; naabdalneim@imamu.edu.sa (N.A.I.); nsbasher@imamu.edu.sa (N.S.B.)

**Keywords:** protein engineering, drug discovery, biologics, monoclonal antibodies, CRISPR-Cas, VHH domains

## Abstract

Protein engineering is a rapidly evolving field that plays a critical role in transforming drug discovery and development. This innovative field harnesses the unique structural and functional properties of engineered proteins, such as monoclonal antibodies, nanobodies, therapeutic enzymes, and cytokines, to address complex diseases more effectively than traditional small-molecule drugs. These biologics not only enhance therapeutic specificity but also minimize adverse effects, marking a significant advancement in patient care. However, the journey of protein engineering is not without challenges. Issues related to protein folding, stability, and potential immunogenicity pose significant complications. Additionally, navigating the complex regulatory landscape can delay the transition from laboratory to clinical application. Addressing these hurdles requires the integration of cutting-edge technologies, including phage and yeast display technology, CRISPR, and advanced computational modeling, which enhance the predictability and efficiency of protein design. In this review, we explore the multifaceted impact of protein engineering on modern medicine, highlighting its potential to transform treatment paradigms, methodologies, challenges, and the successful development and approval of recombinant protein-based therapies. By navigating the complexities and leveraging technological advancements, the field is poised to unlock new therapeutic possibilities, ultimately improving patient outcomes and transforming healthcare.

## 1. Introduction

Protein engineering is a rapidly evolving discipline of molecular biology, biochemistry, immunology, cell biology, and biotechnology, significantly transforming the landscape of drug discovery and development. By modifying protein structures and functions, researchers can design innovative therapeutics that are not only more effective but also safer and better tailored to patient needs. The significance of protein engineering in drug discovery is highlighted by its ability to overcome several limitations characteristic of traditional small-molecule therapeutics [1,2,3]. Historically, small-molecule drugs have formed the backbone of pharmacotherapy; they often face challenges related to specificity, bioavailability, and adverse side effects. However, engineered proteins, such as monoclonal antibodies (mAbs), nanobodies (Nbs), therapeutic enzymes, hormones, vaccines, and cytokines, present unique advantages, and these biologics can be designed to target specific disease pathways with high specificity and effectiveness, thereby reducing off-target effects and enhancing therapeutic efficacy [4,5,6,7,8]. Moreover, the potential for personalized medicine is a compelling aspect of protein engineering, as these engineered proteins can be tailored to the genetic and phenotypic profiles of individual patients, improving treatment outcomes. Despite the potential that protein engineering holds, several critical gaps remain in our understanding of its full potential in drug discovery, and one of the primary challenges is the complexity of protein folding and stability [9,10]. Additionally, precisely predicting how engineered proteins will fold and function in physiological conditions is a significant difficulty, as misfolded proteins can lead to loss of function or even toxic effects, complicating the development process [11,12].

Furthermore, the regulatory landscape for biologics is often complex and lengthy, posing hurdles that can delay the transition from the laboratory to clinical application. The complex requirements for safety and efficacy testing can extend development timelines and increase costs, thereby impacting the possibility of new therapeutics reaching the market [13]. Moreover, numerous success stories exist, and a comprehensive analysis is needed that systematically evaluates the overall impact of protein engineering on drug discovery timelines, costs, and success rates. Understanding these dynamics is crucial for investors, including scientists, clinicians, and policymakers, to make informed decisions about continually investing in and developing protein-engineered-based therapeutics. Furthermore, protein engineering technology has the potential to enhance drug discovery by providing targeted, effective, and personalized therapeutic options. However, significant challenges remain, including technical complexities related to protein expression and purification, structural determination, protein folding, regulatory barriers, and ethical considerations regarding genetic modifications [14,15]. This review aims to provide a detailed overview of the importance of protein engineering in drug discovery, technological advances in production, and efficacy, while elucidating the challenges faced by researchers and outlining prospects for this dynamic field. By addressing these aspects, we hope to contribute to a more comprehensive understanding of how protein engineering can be leveraged to meet the evolving demands of modern medicine and improve patient outcomes.

## 2. Technologies for Structural Determination of Proteins

**Figure 1:**
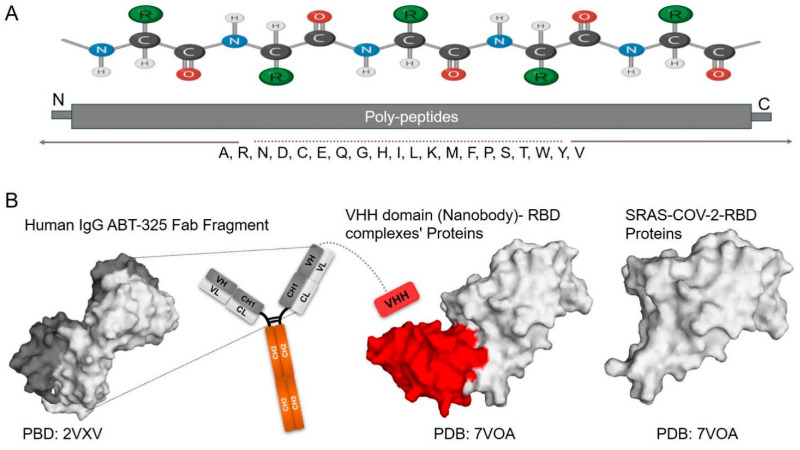
**Protein Structure.** (**A**) A polypeptide chain shows the primary structure of proteins, formed by amino acids linked via peptide bonds. The length and sequence of this polypeptide chain determine the protein’s structure and function. It is composed of various 20 standard amino acids (A, R, N, D, C, E, Q, G, H, I, L, K, M, F, P, S, T, W, Y, V). (**B**) The 3D structures of different proteins, retrieved from the Protein Data Bank (PDB), highlight the structural diversity of proteins: a Human IgG antibody Fab’s fragment (PDB: 2VXV) [21], a Nanobody-SARS-CoV-2 complex (PDB: 7VOA) [22], and the SARS-CoV-2 receptor-binding domain (RBD) protein (PDB: 7VOA). PyMOLv0.99 was used to prepare the figure.

Proteins are highly complex macromolecules composed of one or more polypeptide chains, each made from a sequence of 20 standard amino acids linked by peptide bonds (Figure 1A). The unique sequence and chemical differences in these amino acids, particularly their side chains, determine the physicochemical properties of proteins, such as solubility, stability, and interaction potential [16]. Proteins show a classified structural group: the primary structure (only amino acid sequence), secondary structures (alpha helices and beta sheets stabilized by hydrogen bonds), tertiary structure (the overall three-dimensional conformation), and quaternary structure (assembly of multiple polypeptide chains) (Figure 1B). These structural levels are essential for various biological functions of the protein, and proteins as dynamic macromolecules have the capability of conformational changes in response to environmental factors or ligand binding. These conformational changes are crucial for functional activities, including enzymatic activity, signal transduction, antibody defense, structural support proteins, and transport. Moreover, protein–protein interactions, governed by features such as interface area, hydrogen bonding, and binding energy, are central to cellular processes and can be highly specific accordingly [16,17,18] (Figure 1B). Additionally, the stability and correct folding of proteins are vital for their function, as misfolding can lead to aggregation and diseases such as amyloidosis and neurodegenerative disorders [19]. Remarkably, even proteins with similar three-dimensional (3D) folds may exhibit significant differences in side-chain interactions and accessibility, highlighting the importance of general structural features over specific residue–residue contacts [18]. Advances in technologies such as X-ray diffraction, Cryo-electron microscopy (Cryo-EM), and nuclear magnetic resonance (NMR) enable us to determine the protein structural features and deepen our understanding of their roles. Furthermore, progress in bioinformatic tools now enable the extraction and prediction of protein features from sequence and structure [20].

## 3. Advanced Computational Tools in Protein Engineering

The integration of advanced computational Tools has significantly transformed the field of protein engineering. Technologies like molecular dynamics (MD) simulations, docking studies, and artificial intelligence (AI)-driven approaches are critical for understanding protein behavior, enhancing drug design, and accelerating the development of novel therapeutics. These methods not only explore the complex dynamics of proteins but also predict ligand interactions, improve the efficiency of biological design, and help customize therapeutic proteins for specific clinical needs. MD simulations tools are crucial to understanding the dynamic behavior of proteins at an atomic level. By simulating the movement of atoms and molecules over time, MD allows us to examine how proteins change their structure in response to factors such as temperature and pH, and this is significant for evaluating protein stability and folding pathways, which are crucial for therapeutic effectiveness. For instance, during the SARS-COV-2 pandemic, MD simulations were used to model the SARS-CoV-2 spike protein, identifying important conformational states that enable its interaction with the ACE2 receptor [10,11,23,24]. Consequently, this data was essential for guiding the development of inhibitors that block viral entry, helping in therapeutic strategies and vaccine development. Additionally, in studying protein dynamics, MD simulations have facilitated optimizing therapeutic antibodies. By exploring the effects of mutations on binding affinity and stability, researchers can refine antibody designs to enhance their therapeutic efficacy. For example, a recent study shows that the MD played an essential role in evaluating the stability and binding characteristics of the human epidermal growth factor receptor 2 (HER2)-targeting immunotoxin. The findings support the potential of the designed construct as a therapeutic candidate for HER2-positive breast cancer, paving the way for additional experimental validation through in vitro and in vivo assays [25]. The integration of MD with experimental validation is a powerful strategy for developing next-generation biologics. Docking studies balance MD simulations by predicting the selected orientations of ligands upon binding to proteins, thereby assessing binding affinities and illuminating binding modes. This computational tool is invaluable for high-throughput virtual screening, enabling researchers to rapidly evaluate large libraries of compounds to identify potential drug candidates [26,27]. For example, in the development of small-molecule inhibitors targeting the BCL-2 protein, docking studies facilitated the identification of lead compounds that demonstrated promising efficacy in pre-clinical trials [28].

Moreover, the integration of AI and ML tools into protein engineering has further revolutionized the field. AI-driven approaches leverage vast datasets from structural biology, genomics, and clinical outcomes to identify patterns that inform protein design and therapeutic development [29]. These methodologies allow predictive modeling that analyzes sequence data to predict protein folding and stability, significantly reducing the time required for experimental validation. One of the most distinguished advancements is AlphaFold, which has achieved unprecedented accuracy in predicting protein structures from amino acid sequences [30]. Its application has been involved during the COVID-19 pandemic, providing structural models for various viral proteins and aiding in the design of therapeutic agents and vaccines.

## 4. Biologics: The New Frontier

Biologics, particularly protein-based therapeutics, represent a significant advancement in modern medicine by leveraging the structural and functional differences in proteins to treat various diseases, including cancer, autoimmune disorders, and genetic deficiencies, as well as prevent infectious diseases through vaccines. These therapies include antibodies, fusion proteins, cytokines, and enzyme replacements, all designed to exploit the specificity and high-affinity binding properties characteristic of protein structures [31,32,33]. The effectiveness of biologics stems from their ability to target disease-associated molecules or cells selectively; a feature rooted in the specific amino acid sequences and complex 3D structures of therapeutic proteins. However, the development and clinical use of protein therapeutics face significant challenges, including limited stability, potential immunogenicity, and the need for parenteral administration due to their size and susceptibility to degradation [34]. Remarkably, the developments in protein engineering have enabled the creation of next-generation biologics with improved stability, reduced immunogenicity, and tunable pharmacokinetics, including engineered antibodies and switchable protein therapeutics that allow for controlled activity in vivo [35]. Moreover, delivery systems, such as sustained-release formulations and alternative administration routes like inhalation or subcutaneous injection, are being developed to enhance patient adherence and therapeutic efficacy [36]. Despite these innovations, issues such as protein aggregation, oxidation, and immune responses remain critical considerations in the design and manufacturing of biologic drugs [37]. However, protein-based biologics exemplify the translation of fundamental protein science into transformative therapies, with ongoing research focused on overcoming their unique challenges to maximize clinical benefit.

### 4.1. Antibodies

**Figure 2:**
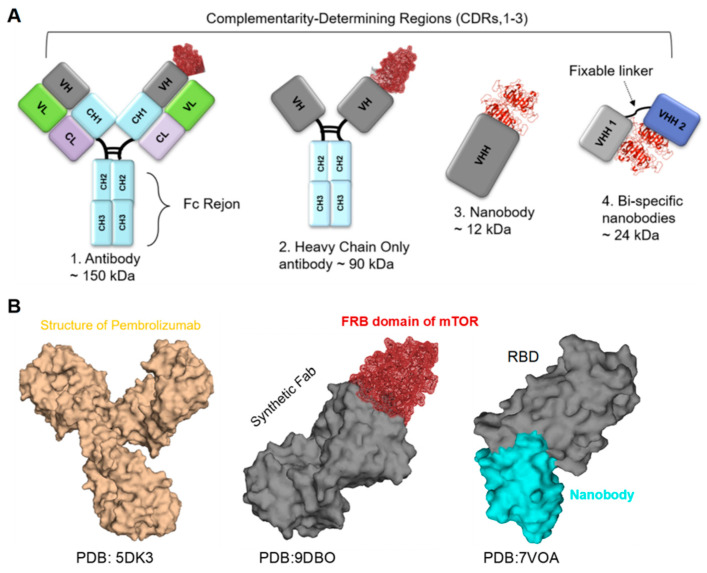
**Antibodies Structure**: (**A**) Shows the structure of a human antibody, highlighting its key components. From left to right (1) full-length Y-shaped Fab region, which is responsible for antigen binding, followed by the Fc region ~150 kDa, (2) Heavy Chain Only ~90 kDa, (3) Nanobody ~12 kDa, (4) Bispecific Nbs ~24 kDa, and this can be engineered to multivalent Nbs and providing more neutralization therapeutic, diagnostic functions [8,22]. (**B**) The 3D structures of various antibodies, which exhibit diverse conformations and varying specificities, underscore the complexity and functionality of antibody design. From left, the structure of a full-length human anti-PD1 therapeutic IgG4 antibody pembrolizumab(PDB: 5DK3) [78], the structure of a synthetic Fab in complex with the FRB domain of mTOR (PDB: 9DBO) [79], and the VHH in complex with SARS-COV-2-RBD (PDB: 7VOA) is represented, demonstrating its unique properties. Numerous studies have shown that the VHH domain is highly effective at neutralization, both as a standalone single domain and in bispecific or multivalent formats [22,65].

Antibodies (Abs), also known as immunoglobulins, are structurally Y-shaped glycoproteins composed of four polypeptide chains: two heavy chains and two light chains. Each antibody has variable regions at the tips of the “Y,” which bind specific antigens, providing high specificity and affinity (Figure 2). The constant regions of the heavy chains determine the antibody class, such as IgG, IgA, and IgM, and mediate several immune functions, including complement activation and binding to Fc receptors on immune cells. Comparison with Nbs, scFv, and fusion Fc-Nbs engineering represents a novel class of therapeutic agents. Nbs consist of a single antigen-binding domain, typically around 12 kDa in size [8] (Figure 2). Another type, single-chain variable fragments (scFv), is an innovative format that comprises the variable regions of both the light and heavy chains, linked by a short peptide, with a molecular weight of approximately 25 kDa [8,38]. Moreover, scFvs retain the specificity of full antibodies while being smaller and easier to engineer. This format allows for the optimization of binding affinity and the potential for fusion with other proteins to enhance functionality. It has been utilized in targeted therapies and diagnostic applications, particularly in cancer immunotherapy and various immunoassay formats. Additionally, fusion Fc engineering involves combining the Fc region of antibodies with additional proteins or peptides, such as scFvs [39] (Figure 2). For example, fusion Fc proteins are particularly valuable in treating autoimmune diseases and cancers, where prolonged therapeutic activity is essential [40,41]. This approach enhances the stability and half-life of the therapeutic agents in circulation while leveraging the effector functions of the Fc region, including complement activation and engagement with immune cells.

However, Abs are therapeutics engineered to recognize and bind to specific antigens, making them powerful tools in treatment and diagnosis. Since their integration, Abs, such as mAb, have become a significant class of biopharmaceuticals, with dozens approved for clinical use and many more in development, reflecting their expanding therapeutic potential and commercial significance [42]. Additionally, their mechanism of action can involve the direct targeting of disease-associated cells, modulation of immune responses, or delivery of cytotoxic agents, and they are valued for their high specificity, reduced off-target effects, and favorable safety profiles compared to traditional small-molecule drugs [43,44]. Moreover, developments in antibody engineering have led to the creation of mAbs with improved pharmacokinetics, reduced immunogenicity, and enhanced efficacy, including modifications that extend their half-lives or enable them to engage immune effector functions more effectively [35]. For example, in oncology, mAbs such as trastuzumab (Herceptin®) validate the targeted approach that has become a hallmark of modern cancer therapy, by specifically inhibiting the HER2 receptor on cancer cells, trastuzumab not only limits tumor growth but also enhances the immune response against these malignant cells [45]. Clinical studies have demonstrated that trastuzumab significantly improves outcomes in HER2-positive breast cancer, leading to its extensive adoption in clinical practice [45,46]. Additionally, mAb, specifically rituximab (Rituxan), targets CD20 on B-cells, effectively treating non-Hodgkin lymphoma and chronic lymphocytic leukemia through both direct cytotoxicity and immune modulation [47]. Furthermore, the development of immune checkpoint inhibitors, such as pembrolizumab (Keytruda®) and nivolumab (Opdivo®), has further expanded the therapeutic landscape, as these agents enhance T-cell responses against tumors by blocking the PD-1 receptor [48,49]. The approval of atezolizumab (Tecentriq), which targets PD-L1, highlights the ongoing advancements in leveraging immune pathways for cancer treatment [50,51]. Moreover, bispecific antibodies (BsAbs) have emerged as a promising strategy, particularly in breast cancer treatment, due to their ability to target two different antigens simultaneously. By targeting tumor-associated antigens (TAAs) on cancer cells, engaging immune effector cells, or blocking critical signaling pathways, BsAbs offer enhanced tumor specificity and increased immune system involvement, thereby improving anti-cancer activity [52,53]. Examples include blinatumomab, which binds to CD19 on cancer cells and CD3 on T-cells, allowing T-cells to recognize and eliminate malignant B-cells. Furthermore, antibody–drug conjugates (ADCs), which combine the specificity of monoclonal antibodies (mAbs) with the potency of cytotoxic drugs, represent another innovative class of anti-cancer compounds widely used in the treatment of hematologic malignancies and solid tumors. By selectively delivering highly potent drugs to tumor cells while sparing healthy tissues, ADCs improve targeted killing of cancer cells and reduce systemic side effects caused by off-tumor toxicity [54,55,56]. Additionally, the emergence of mAbs for SARS-CoV-2 has further demonstrated their therapeutic potential. For example, the combination of casirivimab and imdevimab (REGEN-COV) has been shown to prevent the virus from entering human cells by binding to the spike protein, thereby effectively neutralizing the virus [57]. Additionally, bamlanivimab has demonstrated efficacy in treating SARS-CoV-2 by targeting the spike protein, while sotrovimab has been effective against emerging variants of the virus [58]. However, the effectiveness of some of these mAbs has been questioned due to the emergence of new SARS-CoV-2 variants. Additionally, serological tests utilizing monoclonal antibodies (mAbs) have facilitated the diagnosis of SARS-CoV-2 infections, enabling the rapid identification of prior exposures and supporting public health responses [59,60].

### 4.2. Nanobodies

Nanobodies, also known as single-domain antibodies or heavy-chain-only antibodies (VHH domain), are an innovative class of protein therapeutics derived from camelid species, such as llamas and alpacas. These unique antibodies are approximately 12 kDa, significantly smaller than traditional antibodies, which are typically around 150 kDa (Figure 2). This small size not only facilitates tissue penetration but also enhances binding efficiency to target antigens, making Nbs particularly advantageous for therapeutic and diagnostic applications [8,61]. Their structure, featuring a single variable domain, provides remarkable stability and functionality even under extreme conditions, such as high temperatures and varying pH levels, ensuring continued efficacy and shelf life in clinical settings [8,61,62]. The advancements in clinical research during the SARS-CoV-2 pandemic have significantly increased interest in nanobodies as promising therapeutic and diagnostic agents [8,62]. Nbs have demonstrated the ability to neutralize SARS-CoV-2 by targeting the spike protein and blocking its interaction with the ACE2 receptor, which is essential for viral entry into host cells. Several Nbs have been identified that effectively neutralize SARS-CoV-2 by binding to the RBD of the spike protein, thereby preventing the virus from entering host cells and offering a direct antiviral effect [62,63] (Figure 2). For instance, the Nanosota-1C-Fc nanobody has shown both preventative and therapeutic efficacy in a hamster model of SARS-CoV-2 infection. Additionally, their stability and small size make them suitable for aerosol delivery directly to the lungs, which is particularly advantageous for treating respiratory infections, such as SARS-CoV-2 [64]. Scientists have discovered multivalent Nbs to increase binding avidity and enhance neutralization efficacy against SARS-CoV-2, highlighting their potential in combating the virus [22,65] (Figure 2). Moreover, Nbs have revealed significant potential in treating various infectious diseases, for example, a nanobody that binds to the Ebola virus glycoprotein has demonstrated neutralizing activity in vitro and in vivo, showing potential for therapeutic development [66]. Additionally, Nbs targeting the HIV envelope protein have been discovered as potential therapeutics. The anti-HIV nanobody m36 has been shown to neutralize HIV-1 by binding to conserved regions of the envelope protein, thereby preventing viral entry into host cells. Furthermore, Nbs have been engineered to enhance their binding affinity and broaden their neutralizing capabilities against various HIV strains, illustrating their versatility in combating viral infections [67,68,69]. Another example of Nbs, such as ALX-0171, targeting the RSV fusion protein, has demonstrated effectiveness in treating RSV infections, particularly in infants and young children. This has revealed significant antiviral activity in clinical trials, highlighting the potential of Nbs in treating respiratory infections [70]. Nbs have also been developed against influenza viruses, such as R1a-B6, which exhibits cross-neutralizing activity against different strains of influenza A virus, making it a promising candidate for therapeutic applications [71]. In the realm of Nbs’ applications in cancer therapy, they have demonstrated significant potential in both treatment and diagnostics due to their ability to target tumor-associated antigens with high specificity and effectively penetrate solid tumors. Consequently, Nbs are being assessed as targeting agents to deliver therapeutic radionuclides to tumors; the small size and good tissue penetration features of Nbs make them ideal for delivering cytotoxic molecules to cancer cells [72]. For example, a Phase I clinical trial (NCT02683083) evaluated an ^131^I-labeled anti-HER2 nanobody in breast cancer patients, and nanobody-based diagnostics are being developed for non-invasive cancer diagnosis; they offer advantages such as fast tumor penetration and a short half-life, allowing for same-day molecular imaging with a low radiation load [73,74]. For instance, a Phase I study of a 68Ga-HER2 nanobody demonstrated favorable biodistribution and high accumulation in primary lesions and metastases, with no observed side effects [75]. Advances in bioengineering technology, such as bispecific Nbs, can enhance immune responses and enable the dual targeting of cancer cells, simultaneously targeting tumor-associated antigens and immune effector cells, thereby improving anti-cancer activity. Moreover, Nbs are being utilized as the antigen-binding domain in Chimeric Antigen Receptor (CAR) T-cell therapy; bispecific CAR-T cells constructed with Nbs targeting HER2 and CD20 have shown promising activity against tumor cells [76,77].

### 4.3. Cytokine-Based Therapy

Cytokines are dynamic molecules of the immune system that serve as critical signaling molecules, modulating immune responses and facilitating cell–cell communication. Consequently, they hold significant therapeutic potential in treating cancers, autoimmune disorders, and viral infections. These dynamic proteins are crucial for coordinating the activities of immune cells, including T-cells, B-cells, and macrophages, in response to pathogens and other stimuli [80,81]. Their ability to modulate immune responses makes them essential in maintaining homeostasis and orchestrating the body’s defense mechanisms. Cytokines, based on their functions and the immune responses they mediate, can be categorized into several types, such as interleukins (ILs), tumor necrosis factors (TNFs), interferons (IFNs), growth factors, and chemokines. ILs, are primarily involved in the growth, differentiation, and activation of immune cells, influencing both innate and adaptive immunity, and the second type, TNFs are key regulators of inflammation and apoptosis, playing a significant role in the immune response to infections and tumors [82,83]. Additionally, IFNs are among the most critical cytokines in antiviral defense, enhancing the immune response against viral infections and modulating immune cell activity. Moreover, chemokines act to facilitate the migration of immune cells to sites of infection or inflammation, ensuring an effective and localized immune response [84,85]. Furthermore, Growth factors, such as erythropoietin, stimulate cell proliferation and differentiation, contributing to the overall maintenance of immune system function [86]. However, cytokines hold significant therapeutic potential for treating a range of disorders, including cancers, autoimmune diseases, and viral infections, highlighting their importance in both basic and clinical immunology.

Advancements in protein engineering have enhanced the effectiveness of these proteins through techniques such as mutagenesis and glycosylation modifications, thereby improving their pharmacokinetic properties and reducing side effects. For instance, pegylation of interferon has been functional to increase its half-life and enhance efficacy in clinical trials, and recombinant interferon-alpha, used for the treatment of chronic hepatitis C and certain cancers, demonstrates its impact in drug discovery [87,88]. Moreover, in a study including patients with MAC pulmonary infections, treatment with interferon-γ and chemotherapy showed a significantly better response compared to chemotherapy alone (72.2% vs. 37.5%), with a lower mortality rate in the interferon-γ group (11.1% vs. 35.7%) [89]. However, challenges remain, including the potential for systemic side effects such as flu-like symptoms and fatigue, and immunogenicity, prompting research into targeted delivery systems and localized therapies. Clinical trials have explored combination treatment strategies with various approaches to address these challenges [87,88]. The future of cytokine and interferon therapies is promising, with innovations in synthetic biology, protein engineering, and AI-driven design allowing more effective and personalized treatments. Additionally, collaboration among researchers, clinicians, and industry is essential to translate laboratory discoveries into clinical applications, ensuring these therapies reach patients effectively. Ultimately, the ongoing focus on cytokines and interferons underscores their transformative potential in modern medicine and the evolving landscape of protein therapy.

## 5. Innovations of Protein Engineering-Based Therapies

Recent advances in technology have further revolutionized the field of protein engineering. Methods such as phage display, directed mutagenesis, and CRISPR technology enable high-throughput screening and rapid prototyping of protein variants, allowing scientists to identify promising drug candidates more efficiently. For example, phage and yeast display technologies facilitated the discovery of high-affinity Nbs against challenging targets, and CRISPR technology has rationalized the generation of specific mutations in proteins. Furthermore, the three-dimensional structures of proteins elucidated through advanced structural techniques such as X-ray diffraction and cryo-electron microscopy (Cryo-EM) have significantly enhanced our understanding of the mechanisms underlying several disease pathways. These insights pave the way for protein-engineering-based therapies, leading to the development and approval of numerous engineered and modified proteins for treating and diagnosing challenging diseases. Additionally, the integration of computational modeling and machine learning plays a critical role in enhancing the predictability of protein behavior, enabling researchers to optimize designs before synthesis by screening out genomic libraries or constructed Abs-based libraries. These technological advancements significantly reduce the time and costs associated with protein-based drug development, making the identification process more effective [4,8,48,56,65,69,72,78,90,91,92,93,94,95,96]. In the following sections, we will explore fundamental and advanced methods of protein engineering, including molecular cloning, protein expression using different expression systems, protein purification strategies, and the biochemical and biophysical characterizations of engineered proteins in vitro and in vivo for validation. Our discussion will highlight the strengths and limitations of each method, offering critical insights to help researchers optimize their therapeutic strategies. By equipping scientists with this knowledge, we aim to facilitate the selection of appropriate techniques tailored to specific applications, ultimately advancing the field of protein engineering.

## 6. Methodology

### 6.1. Molecular Cloning

Molecular cloning is a dynamic technique in molecular biology that allows the manipulation, amplification, and expression of specific DNA sequences, and this process is fundamental for the production of recombinant proteins for various applications in research, diagnostics, and therapy. The basic principle of molecular cloning is to insert a gene(s) of interest into a vector, a circular DNA molecule that can carry foreign DNA into a host expression system cell, and the process begins with the selection of an appropriate vector (plasmids, bacteriophages, and artificial chromosomes), each suitable for diverse applications. Plasmids, which are small, circular DNA molecules found in bacteria, are the most commonly used vectors due to their ease of manipulation and their ability to replicate independently within host cells. Bacteriophages, viruses that infect bacteria, are used for cloning larger DNA fragments, such as a library of polyclonal phage, and artificial chromosomes, such as bacterial artificial chromosomes (BACs) or yeast artificial chromosomes (YACs), are designed to accommodate significantly larger inserts, making them suitable for genomic libraries or large gene studies [61,62,65,93,97].

When a suitable vector is selected, the next step is to isolate the DNA fragment of interest, typically via Polymerase Chain Reaction (PCR), restriction enzyme digestion, or Gibson Assembly. The PCR is a powerful technique that amplifies specific DNA to generate sufficient quantities of the target gene(s) from minimal starting DNA templates. Restriction enzymes (REs) can cut DNA at particular sequences based on the type of RE. After digestion with RE, the target DNA is often purified to remove contaminants that could interfere with downstream steps. Following the extraction of the DNA fragment, the next critical step is the insertion of this fragment into the selected vector. This is primarily accomplished by ligation, in which DNA ligase joins the DNA fragment to the digested vector. Furthermore, to ensure efficient ligation, both the vector and the gene(s) are digested with the same RE, creating compatible ends that can be assembled. The ligation reaction must be carefully optimized to achieve high efficiency of the recombinant DNA vector. After constructing the recombinant vector, it is introduced into a host organism by transformation. Standard methods of transformation include heat shock and electroporation. This step is critical for the production and expression of the inserted gene by the host organism’s expression system. In heat shock, competent *Escherichia coli* (*E. coli*) cells are subjected to a dramatic temperature change, making their membranes permeable to the recombinant vector, and electroporation uses an electric field to create temporary pores in the cell membrane, allowing the recombinant vector to enter. Successful transformation allows the host cells to replicate the vector and express the inserted gene accordingly. Transformation is essential for selecting cells that have successfully taken up the recombinant vector. This is typically achieved through antibiotic selection, where the vector contains an antibiotic resistance gene, allowing only those cells that have incorporated the vector to survive in selective media [61]. Screening techniques, such as colony PCR, blue–white screening, or sequencing, are used to identify colonies that contain the desired insert. In blue–white screening, for example, colonies that successfully incorporate the vector with the insert appear white, while those that take up the empty vector turn blue due to the activity of the β-galactosidase enzyme [98]. When positive clones are identified, the next step is to induce protein expression. However, molecular cloning is a powerful and versatile technology that has revolutionized our understanding and manipulation of genetic material. Molecular cloning facilitates the detailed modification and expression of genes and opened new avenues in biotechnology, leading to advancements in therapeutic development, genetic research, and synthetic biology. As techniques continue to evolve, molecular cloning will remain a foundation of biological research and a key driver and fundamental player of innovation in healthcare [99].

### 6.2. Protein Expression Systems

Protein expression systems are essential technology in molecular biology, biotechnology, and pharmaceutical development, permitting the production of recombinant proteins for research, therapeutic, and industrial purposes. These systems can vary widely, with each having unique advantages and limitations that depend on the type of protein, the desired yield, post-translational modifications, and the intended application.

#### 6.2.1. Types of Protein Expression Systems

Protein expression systems are categorized into two main types: prokaryotic and eukaryotic systems, each offering distinct benefits.

(A). **Prokaryotic Systems:** The most commonly used prokaryotic system is *E. coli*. This bacterium is favored for its rapid growth, its well-understood genome, and the simplicity of its culture conditions. *E. coli* can produce high yields of recombinant proteins in a short time frame (4–12 h), making it ideal for producing proteins for research and industrial applications. However, the prokaryotic system has limitations, particularly in post-translational modifications (PTMs), because many eukaryotic proteins require specific PTMs, such as glycosylation, phosphorylation, or other modifications that *E. coli* cannot perform, which can potentially affect protein functionality and stability [8,61].

(B). **Eukaryotic Systems:** Eukaryotic expression systems, such as yeast, insect cells, and mammalian cells, are applied to overcome the limitations associated with *E. coli*. Yeast systems, such as *Saccharomyces cerevisiae*, are cost-effective and suitable for producing proteins that require glycosylation, although the types of glycosylation may differ from those in higher eukaryotes [100]. Furthermore, insect cell systems, particularly using the baculovirus expression vector system (BEVS), allow for the efficient production of complex proteins; the baculovirus can infect insect cells such as *Spodoptera frugiperda*, leading to high levels of protein expression and folding. Furthermore, mammalian cell systems, such as Chinese hamster ovary (CHO) cells or human embryonic kidney (HEK293) cells, are the gold standard for producing therapeutic proteins, because these cells can perform complex PTM similar to those in human cells, resulting in correctly folded and functional proteins. However, mammalian systems are generally more expensive and time-consuming compared to *E. coli* [61,97,101].

#### 6.2.2. Vectors in Protein Expression

Vectors are fundamental tools for cloning, expressing, and manipulating genes; they are engineered to carry the gene(s) of interest into host cells, enabling the production of proteins or the identification of gene function [102]. Understanding the various types of vectors and their distinct features is crucial for achieving successful protein expression and related applications.

#### 6.2.3. Types of Vectors

Vectors can be broadly categorized into three main types: expression vectors, cloning vectors, and delivery vectors. Expression vectors are specifically designed to promote protein expression in host cells; they contain crucial elements such as strong promoters that initiate high transcription levels, ribosome-binding sites for efficient translation, and selectable markers, such as antibiotic resistance genes, to identify successfully transformed cells. Common examples of expression vectors include those based on *E. coli*, yeast, and mammalian systems, each tailored for optimal protein production in its respective host [8,103].

#### 6.2.4. Cloning Vectors

They are primarily used to clone DNA fragments for further analysis or manipulation and these typically feature a multiple cloning site (MCS) that contains several restriction enzyme sites for inserting foreign DNA, along with an origin of replication (ori) that allows the vector to replicate within the host cell, such as pUC19, and pET-30a [61,103,104].

#### 6.2.5. Delivery Vectors

They are crucial in gene therapy, facilitating the introduction of genetic material into target cells; this category includes both viral vectors, which are modified viruses that can efficiently deliver genes, and non-viral vectors, such as liposomes and nanoparticles, that provide alternative methods for gene transfer [105]. The choice of delivery vector often depends on the specific requirements of the therapeutic application, such as the targeted cell type and the desired duration of gene expression.

#### 6.2.6. Key Features of Vectors

Vectors hold several key features that improve their functionality. One of the most important aspects is the presence of selectable markers, which allow researchers to identify cells that have successfully taken up the recombinant vector; common markers include genes that confer resistance to specific antibiotics, which enable the selection of transformed cells when cultured in the presence of the corresponding antibiotic. Additionally, a significant feature is the use of linkers and tags; linkers are short sequences that connect the protein of interest to tags or fusion partners, enhancing solubility and purification of tagged proteins. For example, His-tags, GST-tags, and MBP-tags are commonly used for purification by affinity chromatography (Table 1). Moreover, vectors often include cleavage sites for specific proteases, such as *Tobacco Etch Virus* (TEV) protease, which removes fusion tags after purification, ensuring that the final product is the native protein [50,61,103].

**Table 1:** Examples of Linkers Commonly Used for Protein Fusion.

Linker Type	Linker Name	Sequence	Features	Ref.
Flexible	(Gly4Ser)3	GGGSGGGSGGGSGGGS	Highly flexible; promotes solubility and reduces steric hindrance; commonly used in fusions.	[91,106,107]
Flexible	(Gly)3	GGG	Short flexible linker; enhances solubility; often used in simple fusions.	[107]
Rigid	(EAAAK)3	EAAKEAAKEAAK	Rigid structure; minimizes interaction between fused proteins; functional for complex fusions.	[108,109]
Flexible	Conventional Gly linker	GGGGSGGGG	Provides flexibility with moderate length; suitable for various protein fusions.	[110,111]
Mixed	(Gly/Ser) Linker	GGGSGS	A combination of glycine and serine offers both flexibility and some rigidity.	[112]
Flexible	(Gly)10	GGGGGGGGGG	Very flexible; enhances solubility and minimizes steric clashes, making it suitable for large proteins.	[91]
Rigid	(EAAAK)2	EAAAKEAAK	Rigid; reduces potential steric hindrance; helpful in maintaining structural integrity.	[109]

### 6.3. Therapeutic Protein Purification

**Figure 3:**
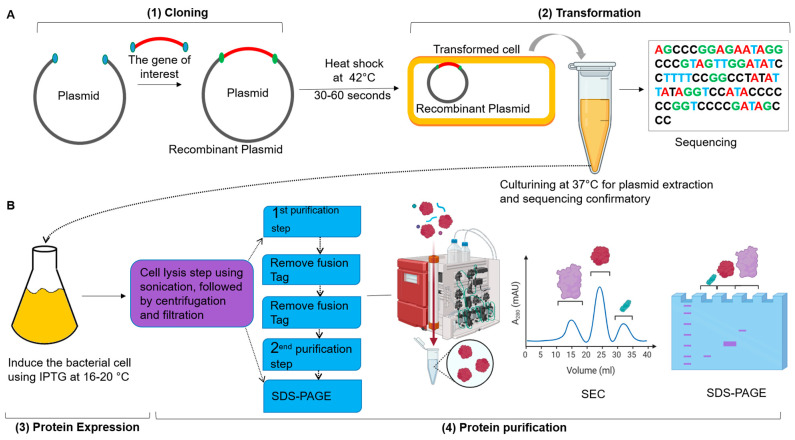
**Cloning, Expression, and Purification of Recombinant Proteins:** (**A**) The workflow begins with molecular cloning, in which the target gene for protein therapy is amplified from the antibody library via PCR. Both the vector and the gene are digested with the same restriction enzymes or assembled using the Gibson Assembly method (1). The transformation is carried out by subjecting the mixture to a heat shock at 42 °C for 30–60 s, followed by incubation at 37 °C for hours in LB medium to grow transformed cells, and then plating on LB agar plates containing specific antibiotics for 24 h. This step facilitates colony PCR and sequencing to verify successful cloning (2). (**B**) Protein expression is induced using Isopropyl β-D-1-thiogalactopyranoside (IPTG) at low temperatures (16–20 °C) to promote proper recombinant protein folding (3). The purification process begins with bacterial cell lysis, followed by centrifugation and filtration to remove cell debris. The resulting cell lysate is then processed through a chelating chromatography column, such as Ni-NTA, to purify the protein. Removal of the tag from the protein is accomplished via dialysis at 4 °C overnight using specific proteases, such as TEV protease. Further purification is achieved through SEC, and Sodium Dodecyl Sulfate–Polyacrylamide gel electrophoresis (SDS-PAGE). Analysis is performed at various stages throughout the purification (4). This protocol for cloning, expression, and purification of recombinant proteins is adapted from [61]. This figure was prepared using BioRender at https://app.biorender.com (accessed on 12 August 2025).

Protein purification technology is a fundamental technique used for isolating specific proteins from complex mixtures. This protease is crucial for studying the structure, function, and interactions of proteins, playing a significant role in applications such as drug and recombinant vaccine development, as well as diagnostics. The techniques of protein purification involve several steps; each is selected according to the properties of the target protein and the intended application. The primary objective of protein purification is to obtain a pure protein in sufficient quantity for further applications. Achieving high purity is essential for downstream applications, including structural studies, enzymatic assays, and therapeutic uses [90]. Purity is critical because the presence of contaminants can interfere with the protein’s functionality and complicate subsequent analyses. Maintaining the integrity of the protein during purification is equally essential, as some methods can denature or modify the protein, resulting in a loss of biological activity [90]. The process of protein purification generally includes several stages, starting with cell lysis, this initial step includes breaking open the cells to release intracellular proteins, which can be accomplished using mechanical disruption, chemical lysis, or enzymatic methods. Following cell lysis, the mixture contains cellular debris; techniques such as centrifugation or filtration are commonly used to clarify the lysate, allowing for the collection of the soluble protein fraction, after obtaining the soluble proteins, an initial fractionation step is often employed, usually involving ammonium sulfate precipitation or dialysis [22,61,103]. Ammonium sulfate precipitation exploits differences in protein solubility at various salt concentrations, enabling the selective precipitation of the target protein while leaving other proteins in solution. This step is critical for concentrating the protein and removing impurities before further purification. Additionally, chromatographic techniques are the backbone of protein purification and can be categorized into several types. For example, His-tagged proteins can be purified using nickel-affinity columns, which offer a high degree of specificity. Affinity chromatography is a highly effective method that utilizes specific interactions between the target protein and a ligand attached to the stationary phase (Table 2). Furthermore, ion exchange chromatography separates proteins based on their charge; in this technique, proteins are applied to a column containing charged resin, allowing them to bind according to their net charge at a specific pH. This method can be beneficial for separating proteins with similar sizes but differing charge properties. Another chromatography technique is Size Exclusion Chromatography (SEC), which separates proteins based on their size; larger proteins elute first, and smaller proteins are retained in the column for a more extended period, facilitating their separation from contaminants. Additionally, hydrophobic interaction chromatography (HIC) exploits the hydrophobic properties of proteins. By manipulating salt concentrations, proteins are encouraged to interact with hydrophobic groups on the stationary phase, allowing for their separation based on hydrophobicity (Figure 3). These chromatographic techniques can be combined in multiple steps to achieve the desired purity [8,62,65,90,103].

### 6.4. Characterization of Recombinant Proteins

Characterization of recombinant proteins is a crucial step in understanding the structure, function, and behavior of these proteins, as they are produced through genetic engineering, where a gene of interest is expressed in a host organism, enabling large-scale production [8,61]. The characterization process provides insights into the protein’s properties, ensuring its suitability for various applications, including therapeutic use, diagnostics, and industrial processes. By carefully analyzing these characteristics, researchers can ensure that the produced proteins meet the necessary conditions for their future applications [113]. The characterization of proteins is a complex process that requires numerous analytical techniques, each with unique strengths and limitations [8,61]. However, most techniques require purified proteins to ensure accurate results, although some methods, such as enzyme activity assays, can analyze proteins directly within complex mixtures, this flexibility is advantageous, allowing for assessments in native contexts, but the purity of the sample can significantly affect reliability [114]. The determination and detection limits of each technique also differ; for instance, X-ray crystallography provides atomic-level resolution, which is invaluable for detailed structural understanding, while techniques like SDS-PAGE offer lower resolution but are faster and simpler for quick assessments of protein size and purity [90]. The selection of method is influenced by the equipment required; basic techniques, such as SDS-PAGE and Western blotting, can be performed with standard laboratory equipment, whereas advanced methods, like NMR spectroscopy and mass spectrometry, require specialized instruments, limiting accessibility for smaller research institutions. The applications highlight the distinct roles that each technique plays across various fields. SDS-PAGE and Western blotting are mainstays in the analysis of recombinant protein expression, while mass spectrometry is critical in proteomics for identifying and characterizing proteins in complex biological samples [115] (Table 2). Additionally, Cryo-EM has emerged as a powerful tool for studying large protein complexes, eliminating the need for crystallization and thereby overcoming a significant hurdle in structural biology [116] (Table 2). Additionally, Isothermal Titration Calorimetry (ITC) and Surface Plasmon Resonance (SPR) are essential techniques for characterizing protein–protein interactions and stability, especially in protein therapy. However, ITC measures heat changes during binding interactions, providing a crucial thermodynamic parameter profile such as binding affinity (kD) and stoichiometry (N), without requiring labeling, making it ideal for studying native protein interactions [61,90]. In contrast, SPR enables real-time, label-free monitoring of recombinant protein interactions, focusing on kinetic parameters such as association and dissociation rates. Its high sensitivity and adaptability to various conditions make SPR a valuable tool for drug design and optimization [65] (Table 2). The selection of ITC or SPR depends on specific research questions and protein characteristics; ITC is well-suited for thermodynamic studies, while SPR is more suitable for kinetic analyses. Scientists should also consider available resources to align their methodology with their study goals [8,22,61,62,65]. Ultimately, the selection of protein characterization techniques should be guided by specific research questions, the nature of the proteins under study, and the available resources. By understanding the strengths and limitations of each method, researchers can make informed decisions that enhance the reliability and relevance of their findings (Table 2).

**Table 2:** Some Examples of Techniques used for Protein Characterization.

Technique	Purpose	Methodology	Applications	Advantages	Disadvantages	Ref.
SDS-PAGE	Separates proteins based on molecular weight	Proteins are denatured and coated with SDS, then subjected to electrophoresis in a polyacrylamide gel	Assessing purity, estimating molecular weight.	Simple, quick, and cost-effective	Does not provide information on protein activity	[61,117,118]
2. Western blotting	Detects specific proteins	Proteins from SDS-PAGE are transferred to a membrane and probed with specific antibodies	Confirming identity and expression levels	Highly specific detection	Requires high-quality antibodies; can be time-consuming	[117,119]
3. Mass Spectrometry (MS)	Analyzes protein mass and structure	Proteins are ionized and fragmented; the mass-to-charge ratio of ions is analyzed.	Identifying proteins, studying modifications	High sensitivity and specificity	Sample preparation can be complex	[120]
4. Enzyme Activity Assays	Measures the functional activity of enzymes	Quantifies the rate of reaction catalyzed by the enzyme using substrates and measuring products	Evaluating enzymatic activity and kinetics.	Directly assesses enzyme functionality.	Requires specific substrates; conditions must be optimized.	[114]
5. Circular Dichroism (CD) Spectroscopy	Analyzes the secondary structure of a protein	Measures the differential absorption of circularly polarized light to assess folding	Assessing protein folding and stability	Quick and requires small sample amounts	Limited to secondary structure; cannot provide 3D structures	[61,121]
6. Nuclear Magnetic Resonance (NMR)	Provides structural information in solution	Detects magnetic properties of atomic nuclei to determine 3D structures and dynamics	Understanding protein conformation and dynamics	Can analyze proteins in solution, retaining the native state	Limited to smaller proteins; requires high concentrations	[122,123]
7. X-ray Crystallography	Determines high-resolution structures	Crystals of proteins are bombarded with X-rays, producing a diffraction pattern that is analyzed for structure	Revealing atomic-level architecture	Provides high-resolution structures	Requires crystallization, which can be difficult	[90,124]
8. Thermal Shift Assays	Assesses protein stability	Measures changes in fluorescence or absorbance as temperature increases	Identifying optimal storage conditions.	Simple and rapid; requires minimal equipment	May not provide detailed stability profiles.	[125,126]
9. Surface Plasmon Resonance (SPR)	Measures real-time interactions	Detects changes in refractive index as proteins bind to ligands on a sensor surface	Studying protein–protein and ligand binding interactions	Real-time measurement of interactions	Requires specific equipment and may need optimization	[61,127,128]
10. Isothermal Titration Calorimetry (ITC)	Measures binding interactions and thermodynamics	Monitors heat changes during binding events to determine affinity and stoichiometry	Analyzing binding interactions of protein–protein and ligands	Provides thermodynamic data in a single experiment	Requires large amounts of protein; can be expensive	[61,129]
11. Cryo-EM	Provides structural information in native states	Samples are rapidly frozen and imaged using electron microscopy to obtain 3D reconstructions	Studying large complexes and membrane proteins	Does not require crystallization; it can study large complexes	Requires specialized equipment and sample preparation	[130,131]
12. Fluorescence Spectroscopy	Analyzes protein conformation and dynamics	Proteins labeled with fluorescent tags are excited, and emission spectra are measured	Monitoring folding and binding events	Sensitive and versatile; can provide dynamic information	Requires labeling; may alter protein behavior	[61,132,133]

## 7. Clinical Cases: Successful Examples

In the rapidly evolving field of medicine, recombinant therapeutic proteins have become a cornerstone in the treatment of various diseases, particularly infectious diseases and cancers. This section explores several clinical cases that demonstrate the successful applications of recombinant therapeutic proteins and innovative therapies in real-world settings. By highlighting these examples, we aim to explain the profound impact of these advanced treatments on patient outcomes and the ongoing evolution of medical practices.

### 7.1. Recombinant Protein Fighting Infectious Diseases

Recombinant proteins have become energetic in the treatment of infectious diseases, particularly through mAb therapies, such as Bamlanivimab, a monoclonal antibody developed by Eli Lilly, which received Emergency Use Authorization (EUA) for the treatment of SARS-COV-2 [134] (Table 3). In clinical trials, it demonstrated a significant reduction in viral load and hospitalization rates for high-risk patients. Additionally, Casirivimab and Imdevimab, another distinguished example, is a combination of Casirivimab and Imdevimab, which also received EUA for COVID-19. This cocktail therapy exhibited promising efficacy in reducing the risk of hospitalization and death in non-hospitalized patients with mild to moderate SARS-COV-2 [135,136] (Table 3).

### 7.2. Recombinant Protein as Cancer Therapy

Monoclonal antibodies play a crucial role in oncology, offering targeted therapies that significantly enhance treatment outcomes. Trastuzumab (Herceptin) targets the HER2 receptor, which is overexpressed in certain types of breast cancer. In crucial clinical trials, it improved survival rates and reduced recurrence in HER2-positive breast cancer, leading to its approval and widespread use [137,138] (Table 3). Additionally, pembrolizumab (Keytruda); Pembrolizumab, an anti-PD-1 mAb, is used to treat various cancers, including melanoma and non-small-cell lung cancer, and the clinical studies have confirmed its ability to enhance the immune response against tumors, leading to significant improvements in progression-free survival [139,140] (Table 3).

### 7.3. Cytokine Therapies

Cytokines have been successfully engineered to enhance immune responses against various diseases. For example, Interferon-α (INF-α) has been a cornerstone in treating chronic hepatitis C, as it has been shown to enhance the immune response and decrease viral loads [141]. The introduction of pegylated interferon has improved patient compliance and treatment outcomes significantly [142]. Additionally, interleukin-2 (IL-2) (Aldesleukin, a recombinant form of IL-2) has been used to treat metastatic renal cell carcinoma and melanoma. It stimulates T-cell and natural killer cell activity, resulting in durable responses in some patients [143,144].

### 7.4. CAR T-Cell Therapy

CAR T-cell therapy represents a novel approach to cancer treatment, utilizing genetically modified T-cells, this innovative therapy involves regulating a patient’s T-cells, enabling them to target and destroy cancer cells specifically [145]. One prominent example of this therapy is Kymriah (tisagenlecleucel), which is specifically designed to target CD19-positive B-cells [146]. This treatment has shown remarkable efficacy, particularly in pediatric patients with acute lymphoblastic leukemia (ALL) [147]. Additionally, notable CAR T-cell therapy is Yescarta (axicabtagene ciloleucel), which also targets the CD19 antigen but is explicitly approved for adult patients diagnosed with large B-cell lymphoma [148,149]. Many patients treated with this therapy have experienced complete remission, these findings underscore the transformative potential of CAR T-cell therapies in the treatment landscape for various blood cancers, offering hope to patients who may have limited treatment options [150,151,152]. The efficacy of these therapies continues to be a focal point in ongoing clinical research and trials, as shown in Table 3.

### 7.5. Nanobodies in Disease Treatment

Nbs offer distinct advantages in targeting specific antigens, particularly in oncology and autoimmune diseases, such as rheumatoid arthritis (RA). The first Nanobody® therapeutic for RA is Ozoralizumab (Nanozora), a novel TNF inhibitor approved in Japan in September 2022. This next-generation nanobody utilizes two human TNFα-binding domains and a human serum albumin-binding domain, which enhances its plasma half-life and allows for administration every four weeks [153]. Clinical studies have demonstrated remarkable improvements in clinical symptoms and patient-reported outcomes within two days of subcutaneous administration of 30 mg Ozoralizumab, particularly when used alongside methotrexate therapy. The drug’s efficacy and tolerability have been confirmed for up to 52 weeks, making it a promising new option for RA patients seeking effective treatment with early symptom relief [153,154,155]. In addition to Ozoralizumab, other therapies for cancer treatment, such as caplacizumab, a nanobody targeting von Willebrand factor (VWF), have been investigated for their efficacy in treating acquired thrombotic thrombocytopenic purpura (aTTP) [156,157]. Additionally, Bispecific CAR T-cell therapy LCAR-B38M has demonstrated significant efficacy in a multicenter clinical trial in patients with advanced relapsed/refractory multiple myeloma (RRMM) [158] (Table 3).

**Table 3:** Examples of Approved Recombinant Protein Therapies, CAR T-Cell Therapies, and Nanobodies in Clinical Use and Trials.

RPT*^1^ Name	Target Diseases	Technique	Mechanism of Action	Ref.
Recombinant Insulin	Diabetes Mellitus	rDNA*^2^	Regulates blood sugar levels	[159]
Erythropoietin (EPO)	Anemia	rDNA	Stimulates red blood cell production	[160,161]
Adalimumab (Humira®)	Rheumatoid arthritis	rDNA	Inhibits TNF-alpha	[162]
Nivolumab (Opdivo®)	Melanoma, lung cancer	rDNA	Blocks PD-1 receptor to enhance immune response	[49,163]
Bevacizumab (Avastin®)	Various cancers (e.g., colorectal cancer)	rDNA	Inhibits VEGF to prevent tumor blood supply	[164]
Tocilizumab (Actemra®)	Rheumatoid arthritis, COVID-19	rDNA	Inhibits IL-6 receptor	[165]
Atezolizumab (Tecentriq®)	Various cancers (e.g., hepato cellular cancer)	rDNA	Blocks PD-L1 to enhance the immune response	[166,167]
Rituximab (Rituxan®)	Non-Hodgkin lymphoma	Hybridoma technology	Targets CD20 on B-cells	[168,169]
Blinatumomab (Blincyto®)	Acute lymphoblastic leukemia	Bispecific T-cell engager (BiTEs)*^3^	Engages T-cells to target leukemia cells	[170,171]
Tisagenlecleucel (Kymriah®)	Acute lymphoblastic leukemia	CAR T-cell therapy	Redirects T-cells to target leukemia cells	[172,173]
Axicabtagene Ciloleucel (Yescarta®)	Large B-cell lymphoma	CAR T-cell therapy	Redirects T-cells to target lymphoma cells	[174]
Zanubrutinib (Brukinsa®)	Chronic lymphocytic leukemia	Small molecule inhibitor	Inhibits BTK to reduce B-cell activation	[175,176]
Ciltacabtagene	Multiple Myeloma	Phage display technology	Targets the B-cell maturation antigen (BCMA) on multiple myeloma cells.	[177]
Ablynx ALX-0171 (nanobody)	Respiratory syncytial virus (RSV)	Phage display technology	Binds to RSV F protein to neutralize the virus, effectively inhibited replication below the detection limit for 87% of the tested viruses	[178]
Caplacizumab (Cablivi®)	Thrombotic thrombocytopenic purpura (TTP)	Phage display technology	Inhibits von Willebrand factor (vWF)	[179,180]
aSA3-Fc (nanobody)	SARS-COV-2	Phage display Technology	Binds and potently neutralizes SARS-CoV-1, 2, and Omicron; in pre-clinical trials	[65]

RPT*^1^: Recombinant Protein Therapy, rDNA*^2^: Recombinant DNA technology, and BiTEs*^3^: Are engineered proteins that can simultaneously bind to two different targets. Typically, one arm of the BiTE binds to a specific antigen on the surface of cancer cells, while the other arm binds to CD3, a component on T-cells. BiTEs represent a novel strategy in cancer therapy, aiming to improve outcomes through targeted immune response.

## 8. Challenges and Prospects

Protein engineering faces several critical challenges that must be addressed to unlock the full potential of recombinant proteins. One of the primary challenges is the complexity of protein folding and stability; accurately predicting how engineered proteins will fold and function in physiological conditions remains a substantial difficulty, and misfolded proteins can lead to loss of function or even toxic effects [181]. Studies have highlighted the role of evolutionary processes in selecting amino acids that result in stable proteins. Interestingly, advancements in artificial intelligence (AI), such as AlphaFold2, are being utilized to understand how mutations affect protein stability, a crucial factor in maintaining function and preventing disease. However, AlphaFold’s limitations stem from its training on stable proteins, which can lead to predictions that may not account for potential instability [182,183].

The regulatory landscape for recombinant proteins presents another significant challenge. Complex and lengthy regulatory processes often delay the transition from laboratory discoveries to clinical applications [184]. Moreover, safety and efficacy testing requirements extend development timelines and increase costs, requiring a comprehensive understanding of both scientific and regulatory landscapes [185]. Additionally, Immunogenicity is a critical concern, as engineered proteins may provoke immune responses that can reduce their effectiveness and lead to adverse outcomes. For example, in clinical studies of the FDA-approved nanobody caplacizumab, immunogenicity was observed in 3% of patients without impacting clinical efficacy [186]. Understanding the mechanisms of immunogenicity is crucial for developing precise methods to measure immune responses and designing novel particles that can overcome immune-modulatory effects. Other examples, like recombinant human erythropoietin, pose risks such as stroke or myocardial infarction when treatment aims for normal hemoglobin levels [187,188].

Additionally, the effective delivery of biologics to target cells remains a significant challenge, especially for larger recombinant proteins that must cross cellular membranes. Consistently, achieving targeted delivery is crucial for maximizing therapeutic efficacy, as recent advancements have introduced intracellularly targeted biologics for various therapeutic areas, including oncology and genetic disorders. Delivery methods may include physical approaches, direct engineering with targeting moieties, or synthetic and biological nanocarriers [189,190,191]. Moreover, high costs and lengthy timelines associated with developing protein-based therapeutics limit the feasibility of bringing innovative drugs to market. Additionally, the financial implications of clinical trial delays are significant and require careful examination, and a broader estimate account for a variety of costs associated with clinical trial delays, including operational expenses, regulatory compliance, and missed market opportunities, as well as potential loss of sales revenue. This difference highlights the multifaceted financial implications of delays in the drug development process. Other challenges, such as regulatory approvals and ethics committee reviews, can add time-consuming layers, potentially extending timelines for new therapies. Despite these challenges, the prospects for protein engineering are promising. Advancements in computational techniques, including machine learning and AI-driven design, may improve the predictability of protein behavior, accelerating the drug discovery process [192]. Additionally, personalized medicine offers opportunities to tailor engineered proteins to individual genetic profiles, enhancing treatment outcomes and minimizing adverse effects [193]. Moreover, next-generation biologics, characterized by improved stability, reduced immunogenicity, and tunable pharmacokinetics, demonstrate innovative progress in the field [194]. Combination therapies, particularly in oncology, are gaining traction, utilizing engineered proteins alongside other treatments to enhance efficacy and overcome resistance mechanisms [195,196]. Furthermore, quality concerns in protein engineering necessitate maintaining consistency and reproducibility in production and therapeutic efficacy. Variability in manufacturing processes can lead to differences in outcomes, necessitating stringent quality control measures. Characterization of recombinant proteins is crucial to ensure functionality and safety, as inadequate characterization may result in unexpected complications during clinical applications [197,198]. Continuous monitoring of immunogenic responses is essential for identifying potential adverse effects early in clinical trials, necessitating advanced analytical techniques to assess immune responses accurately [34,199]. However, the field of protein engineering and drug discovery faces significant challenges; promising opportunities are on the horizon. By addressing these challenges and leveraging emerging prospects, the field can advance, ultimately leading to improved therapeutic options and enhanced patient outcomes.

## 9. Conclusions

Protein engineering has fundamentally transformed drug discovery, offering innovative strategies for developing targeted therapies that address complex diseases, including cancer, autoimmune disorders, and viral infections. This review has highlighted the pivotal role of engineered proteins, particularly monoclonal antibodies and nanobodies, in enhancing therapeutic efficacy and safety by overcoming the limitations associated with traditional small-molecule drugs. Despite the significant advancements, challenges persist in the field, particularly regarding protein folding, stability, and immunogenicity. These factors can complicate the development and clinical application of engineered proteins. Additionally, the regulatory landscape for biologics is often intricate, posing hurdles that can delay the introduction of new therapies to market. To address these challenges, the application of advanced methodologies such as molecular cloning, CRISPR-Cas technology, and directed mutagenesis is essential. Molecular cloning enables precise manipulation of DNA sequences, enabling the production of recombinant proteins with enhanced properties. This technique facilitates the insertion of genes into vectors, promoting their expression in various host systems, including bacteria, yeast, and mammalian cells. CRISPR-Cas technology has revolutionized gene editing by providing a powerful tool for targeted modifications in protein-coding sequences. This approach enables the identification of specific mutations that can enhance protein stability, alter binding affinities, or improve therapeutic efficacy. Additionally, techniques such as directed mutagenesis enable researchers to introduce precise changes in amino acid sequences, further refining the functional attributes of engineered proteins. The integration of these cutting-edge methods not only accelerates the development of novel biologics but also paves the way for personalized medicine, where therapies can be tailored to individual genetic profiles, thereby improving treatment outcomes. Collaboration among researchers, clinicians, and industry investors is crucial for translating laboratory advancements into clinical applications. Continuous investment in research and development, along with robust regulatory frameworks, will be vital in navigating the complexities of protein-based therapeutics.

However, the future of protein engineering is promising, with the potential to revolutionize healthcare through innovative and effective treatment options. By addressing existing challenges and leveraging emerging opportunities, the field can make significant advancements, ultimately leading to enhanced therapeutic solutions and improved patient outcomes worldwide. The path ahead is one of discovery and collaboration, indicating a new era in the fight against diseases that have long posed challenges to the medical community.

## Data Availability

No new data were created or analyzed in this study.
